# miR-222 expression is correlated with the ATA risk stratifications in papillary thyroid carcinomas∗

**DOI:** 10.1097/MD.0000000000016050

**Published:** 2019-06-21

**Authors:** Dapeng Xiang, Bin Tian, Tianyao Yang, Zhiyu Li

**Affiliations:** a Department of General Surgery, Second Affiliated Hospital School of Medicine, Zhejiang University, Hangzhou, Zhejiang Province, China; b Department of General Surgery, Zhuji Central Hospital, Shaoxing, China; c Tiantai People's Hospital of Zhejiang Province, 317200, China.

**Keywords:** ATA risk stratification, miR-222, papillary thyroid carcinoma

## Abstract

**Background:**

miR-222 is one of the most consistently overexpressed miRNAs in papillary thyroid carcinoma (PTC). Previous studies demonstrated that miR-222 overexpression conferred high-risk features in PTC patients, suggesting its value in risk-stratification. However, studies in term of miR-222's utility on stratifying PTCs are lacking.

**Methods:**

One hundred patients including 10 with multinodular goiter and 90 with PTC were enrolled. Formalin-fixed paraffin-embedded samples were exploited for miR-222 quantitative reverse transcriptase- polymerase chain reaction (RT-PCR) analysis. Correlations between miR-222 expression and different clinicopathological features, Tumor-node-metastasis (TNM) staging and ATA risk level were analyzed.

**Results:**

miR-222 expression of the PTC group was significantly higher than that of the goiter group (*P* < .001). Furthermore, miR-222 expression was significantly higher in PTCs with advanced features like larger tumor, capsular invasion, vascular invasion and lymph nodes metastasis. The majority of patients (61%) were in stage I group (similar to ATA low-risk) by TNM staging system. As to the ATA system, the majority (73%) were in intermediate-risk group (similar to TNM stage II and III roughly). Contrary to previous report, here we found that miR-222 expression was correlated with the ATA risk level (*P* < .001), but not with the TNM staging (*P* = .122).

**Conclusion:**

In the present study, we demonstrated that miR-222 overexpression was correlated with advanced features like capsular invasion, vascular invasion, larger tumor size and lymph node metastasis in PTCs. Most importantly, miR-222 expression was correlated with ATA risk levels, suggesting its potential value in PTC risk-stratification.

## 1 Introduction

Papillary thyroid carcinoma (PTC) is the most common form of thyroid carcinomas. With the prevalence of ultrasound, increasing numbers of new cases are detected each year.^[1]^ Most PTCs are indolent and have an excellent prognosis. However, recurrences are observed in approximately 40% of PTC patients.^[2,3]^ A small portion of PTCs with aggressive types were insensitive to radioiodine treatment after thyroidectomy and showed relative high rates of tumor-related mortality.^[4–7]^

Post-operative risk-stratification is recommended for all patients with PTC to provide prognostic information for disease surveillance and therapeutic strategies making. Various biomarkers have been developed for better prognosis prediction. However, few biomarkers have been shown to be sensitive and reliable enough in tumor staging, rendering the majority of them not been incorporated into the current staging systems.^[8]^

MicroRNAs (miRNAs) are short endogenous non-coding molecules that regulate gene expression at the post-transcriptional level and aberrant miRNA expression can activate multiple oncogenic pathways in different forms of cancer.^[9]^ miR-222 is one of the most consistently overexpressed miRNAs in PTCs. An increase of 11 to 19 fold compared to normal thyroid tissues has been reported.^[10]^ Previous studies showed that miR-222 had a high sensitivity in diagnosing thyroid malignancies.^[11]^ Besides, overexpression of miR-222 along with other miRNAs was correlated with high-risk features such as extrathyroidal extension, lymph node metastasis, distant metastasis and recurrence in PTCs.^[12–15]^ These studies implied that miR-222 could be potentially used as one biomarker for PTC stratification. However, studies about the utility of miR-222 on risk stratification are still limited. In this study, we aimed to find out whether miR-222 expression was correlated with the ATA risk stratification (2009 edition) in PTCs and tried to examine its clinical value as a biomarker for PTC stratification.^[16]^

## 2 Materials and methods

### 2.1 Patients

The study design and protocol were approved by the Ethics Committee of Second Affiliated Hospital, Zhejiang University College of Medicine. From January 2014 to May 2015, 90 consecutive PTC patients undergoing thyroidectomy with central neck dissection were included for this study. In this period, 10 patients underwent thyroidectomy for multinodular goiter were selected as the benign control group. Data regarding patient demographics and primary tumor histopathology (tumor size, lymph node metastasis, TNM staging and ATA risk stratification) were obtained by reviewing the patients’ medical records.

### 2.2 Specimens

Formalin-fixed paraffin-embedded (FFPE) tissues samples were obtained at the Department of Pathology, Second Affiliated Hospital, Zhejiang University College of Medicine. All tumors were reviewed by two pathologists. For FFPE tissues, tumor targets were manually micro-dissected from two to four 10 μm histologic sections for RNA extraction. The total amount of RNA was extracted using miRNeasy FFPE kit (Qiagen, Valencia, CA) according to manufactures protocol. RNA yield was determined using the NanoDrop 1000 spectrophotometer (ThermoScientific, Wilmington, DE).

### 2.3 miRNA quantitative RT-PCR

The measurement of the expression levels of individual miRNAs was performed by the real time-RT-PCR-based detection methodology. Endogenous small RNA U6 was used as endogenous control for the normalization of RNA input. Forward and reverse primers are demonstrated as follows: Forward Primer (miR-222): 5’-GTTCGTGGGAGCTACATTGTCTGC-3’, Reverse Primer (miR-222): 5’-GTGTCGTGGAGTCGGCAATTC-3’. Forward Primer (U6): 5’-GCTTCGGCAGCACATATACTAAAAT-3’, Reverse Primer (U6): 5’-CGCTTCACGAATTTGCGTGTCAT-3’. All reactions involved initial denaturation at 95°C for 30 seconds followed by 40 cycles of 10 seconds at 95°C, 20 seconds at 60°C and 34 seconds at 72°C. All RT-PCR reactions were performed in triplicate. miRNA expression levels were calculated by relative quantitation using the ABI 7500 Real-Time PCR SDS 1.2 software (Applied Biosystems Inc., Foster City, CA). Mean Ct values were calculated for each specimen and then normalized against the corresponding U6 Ct values, calculated as (Ct_miR-222_−Ct_U6_). All data presented were normalized 2^−ΔCt^ values.

### 2.4 Statistical analysis

Statistical analysis was performed using the SPSS 17.0 statistical package program. All statistical analyses were performed using the Mann-Whitney *U* between 2 groups or Kruskal-Wallis *H* test among three or more groups. *P* < .05 was considered as statistically significant.

## 3 Results

### 3.1 Demographics

There were 100 patients enrolled in this study, including 90 with PTC and 10 with multinodular goiter. All 10 patients with multinodular goiter underwent subtotal thyroidectomy. Demographics and pathologic tumor features of 90 PTCs are listed in Table 1.

**Table 1:**
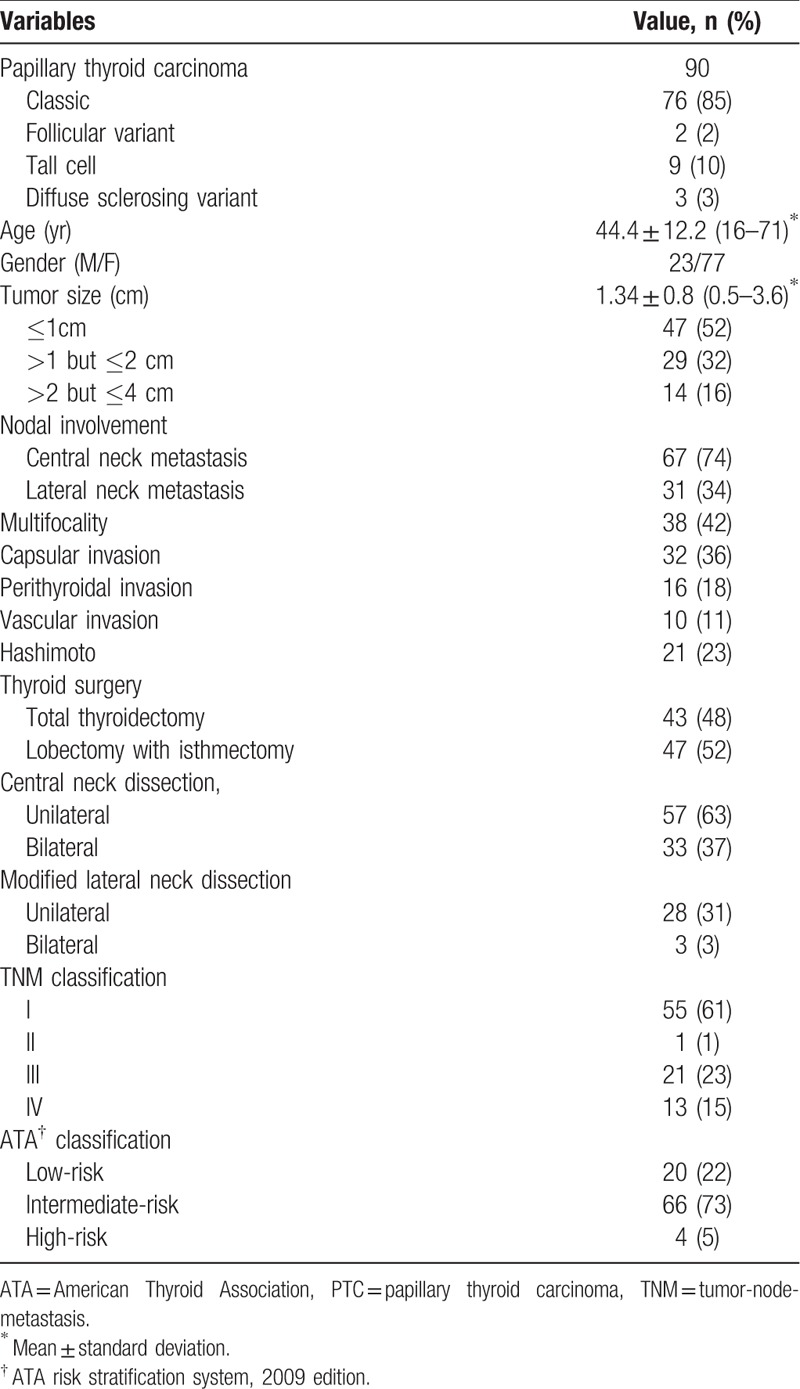
Demographics and clinical characteristics of 90 PTCs.

Of the 90 PTC patients, there were 77 female (86%) and 23 male (14%) patients with a mean age at diagnosis of 44.4 years (range 16–71 years). 43 patients were < 45 years old and 47 patients were ≥45 years old. The average tumor diameter was 1.34 cm (range 0.5–3.6 cm). Forty-seven patients (52%) had microcarcinomas (≤1 cm). Twelve PTCs showed aggressive histology with 9 tall cell and 3 diffuse sclerosing variant. All 90 PTC patients underwent total thyroidectomy with central neck dissection. Thirty-one PTC patients underwent lateral neck dissection simultaneously. Sixty-seven patients (74%) had central neck metastasis (CNM) and 31 patients (34%) had lateral neck metastasis (LNM). According to the TNM staging system, 55 patients (61%) were classified as stage I. Twenty-two patients (24%) in total were classified as stage IIand III (similar to intermediate-risk group by ATA classification). Thirteen patients (15%) were classified as stage IV. As to the ATA system, 20 patients (22%) were classified as low-risk. Sixty-six patients (73%) were classified as intermediate-risk. Four patients (5%) were classified as high-risk.

### 3.2 miR-222 relative expressions according to different clinicopathological characteristics

**Figure 1:**
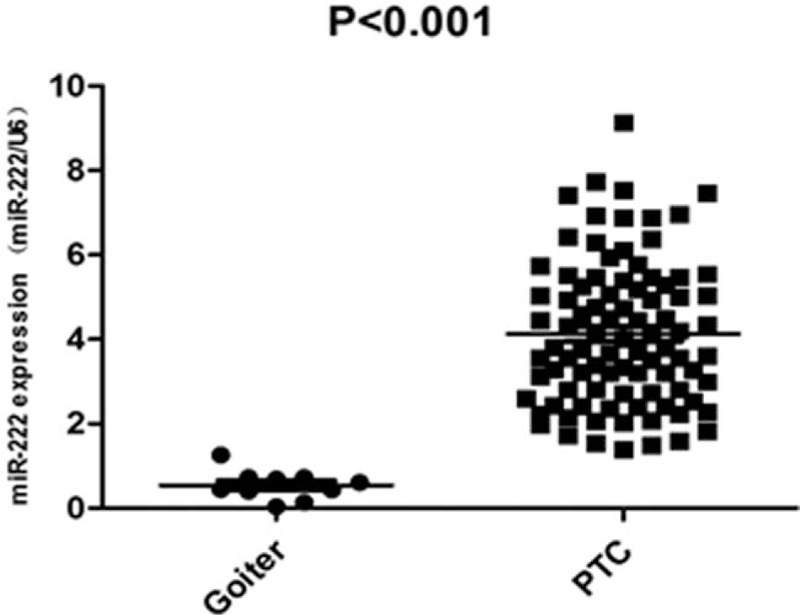
miR-222 expressions of the benign group and the PTC group. Goiter: 10 specimens of multinodular goiter. PTC: 90 specimens of PTCs, including 76 classic type, two follicular variant, nine tall cell and 3 diffuse sclerosing variant.

As expected, the miR-222 expression of the PTC group was significantly higher than that of the goiter group (*P* < .001, Fig. 1). We then analyzed miR-222 expression of the 90 PTCs according to different clinicopathological characteristics (Table 2). Firstly, the miR-222 expression was correlated with the tumor size (*P* < .001). PTCs with a largest tumor diameter >2 cm showed the highest mean miR-222 level. Besides, the miR-222 expressions were significant higher in PTCs with capsular invasion and vascular invasion (*P* = .033 and *P* = .006, respectively). Furthermore, the miR-222 expressions were significantly higher in PTCs with CNM as well as LNM (*P* < .001 and *P* < .001, respectively). The mean miR-222 expression was higher in PTCs with LNM than CNM, although the difference was not significant (5.64 vs 5.15, *P* = .18). Finally, miR-222 expression was correlated with ATA risk stratification (*P* < .001), but not with TNM staging in the present study (*P* = .12, Table 2).

**Table 2:**
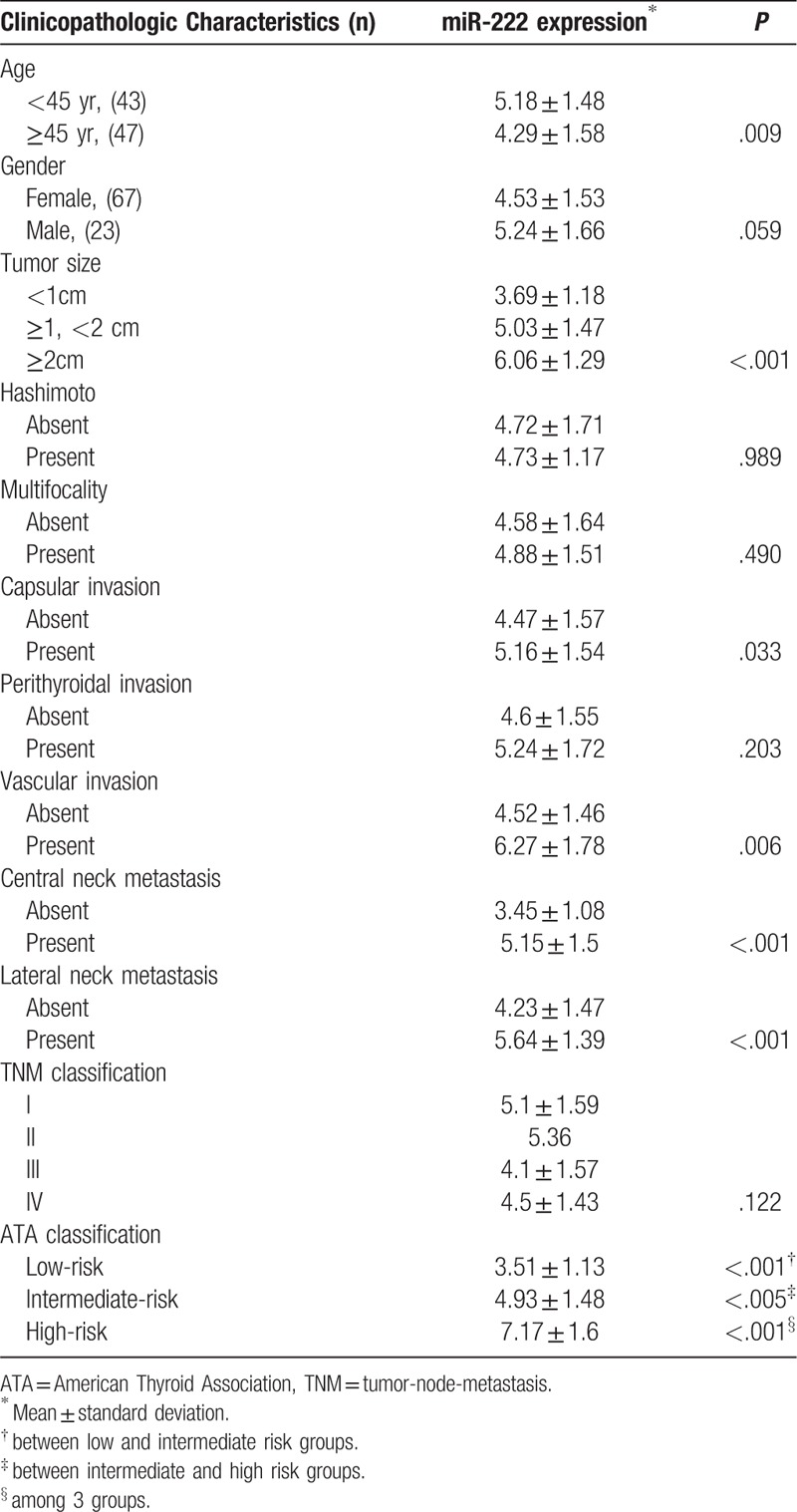
miR-222 expression of 90 papillary thyroid carcinomas.

## 4 Discussion

The majority of PTCs are indolent with excellent prognosis. However, a small portion with aggressive clinicopathological features showed unfavorable outcomes.^[6,17]^ Accurate stratification for patients with PTC is essential to avoid overtreatment of the majority and to provide adequate management for the minority with aggressive disease. No PTC staging systems incorporated biomarkers or molecular testing results until the recent 2015 ATA guidelines suggested a coexistence of BRAF and TERT mutations for PTCs with less favorable outcome. Therefore, efficient molecular markers are still urgently needed for accurate risk-stratification.^[8,18]^

Since He et al^[10]^ reported the preliminary evidence of a potential role for miRNAs in PTC, more deregulated miRNAs have been identified in different types of thyroid carcinomas.^[19–22]^ Of note, overexpression of miR-222 was consistently observed in PTCs, indicating its onco-miRNA role in tumor progression.^[21]^ In the past 10 years, abundant evidences have shown that miR-222 overexpression was correlated with high risk features like extrathyroidal extension, aggressive PTC types, lymph node metastasis, distant metastasis and recurrence in PTCs.^[12–15,23]^ A most recent study found that in classical PTCs, miR-222 is an independent predictor of CNM.^[24]^ These results indicate a great potential of miR-222 in risk stratification.

In accordance with previous reports,^[14,24]^ our study also demonstrated that miR-222 was significantly overexpressed in the subgroups with advanced features like capsular invasion, larger tumor size and lymph node metastases. Further to Aragon et al’ study,^[24]^ we found that miR-222 overexpression was correlated with CNM as well as LNM. Vascular invasion is one important pathology factor for risk stratification and weighs more in the latest ATA system. Of note, our study showed that miR-222 was significantly overexpressed in the subgroup with vascular invasion (*P* = .006). Finally, to integrate all these clinicopathological characteristics and analyze their comprehensive correlation with miR-222 expression, we exploited 2 widely used systems, the AJCC/UICC TNM staging and ATA risk stratification system. Contrary to previous report,^[14]^ we could not find any correlation between miR-222 expression and TNM system (*P* = .122). However, miR-222 expression was indeed significantly different among three risk-stratified groups by ATA system, with miR-222 most expressed in the high-risk group and least expressed in the low-risk group (*P* < .001).

Of note, we also found miR-222 level of young age patients (<45 years) was significantly higher than that of the older age group (≥45 years) (*P* = .009). This could be partially explained by that there were more PTCs with advanced features in the young age group. Actually, we believe this was also the reason why miR-222 expression was not correlated with the TNM staging. In this study, all 43 young patients were uniformly classified as TNM stage I, regardless of any advanced features like capsular invasion, vascular invasion or aggressive histology. The patient distribution also showed the difference that, by ATA stratification 73% of the patients were intermediate-risk and only 22% were low-risk. On the contrary, 61% of the patients were in stage I group (similar to ATA low-risk) and only 22% in total were in stage II and III (similar to ATA intermediate-risk roughly) by TNM staging.^[25]^

Although not correlated with miR-222 expression in this study, TNM system is still the best mortality predicting system with the highest proportion of variance,^[26,27]^ thus is strongly recommended for all patients with PTC according to the ATA guidelines.^[8]^ Besides, a high miR-222 expression does not necessarily suggest a more aggressive histopathology type or one intensive therapy. In the present study, several ATA low-risk cases indeed showed relatively high miR-222 expression than the intermediate-risk ones. This relative inability to accurately stratify the risk for an individual patient with PTC may be related to the failure of current staging systems to adequately integrate other potentially important molecular profiles.^[8]^ However, our study showed that miR-222 expression combined with other clinicopathological risk factors could provide useful information for risk stratification and follow-up decision making.

There were several limitations in present study. First, this was a retrospective study and selection bias could not be totally excluded. Secondly, PTC patients with recurrence or distant metastasis were not included in present study because no qualified specimens were available. A long-term follow-up of the patients and studies with larger samples are necessary to prove the efficiency of miR-222 in PTC risk stratification.

In conclusion, in the present study we demonstrated that miR-222 was overexpressed in PTCs and was correlated with advanced features like capsular invasion, vascular invasion, larger tumor size and lymph node metastasis. Most importantly, miR-222 expression was correlated with ATA risk level but not to TNM staging. Our study suggested that miR-222 may be useful in PTC stratification.

## Acknowledgments

We thank 2 pathologists (Department of Physiology, School of Medicine, Zhejiang University, Hangzhou, China) for their contribution.

## Author contributions

**Conceptualization:** Zhiyu Li.

**Data curation:** Dapeng Xiang, Bin Tian, Tianyao Yang.

**Funding acquisition:** Zhiyu Li.

**Investigation:** Bin Tian, Tianyao Yang.

**Methodology:** Bin Tian, Tianyao Yang.

**Project administration:** Zhiyu Li.

**Resources:** Dapeng Xiang.

**Software:** Dapeng Xiang.

**Supervision:** Dapeng Xiang, Zhiyu Li.

**Validation:** Dapeng Xiang.

**Writing – original draft:** Dapeng Xiang, Bin Tian.

**Writing – review & editing:** Dapeng Xiang, Zhiyu Li.
